# Ruptured Subcapsular Liver Hematoma: A Rare Complication of HELLP Syndrome

**DOI:** 10.1155/2020/8836329

**Published:** 2020-09-16

**Authors:** Pratishtha Singh, Kayle Warren, Victor Collier

**Affiliations:** Department of Internal Medicine, Grand Strand Medical Center, Myrtle Beach, SC, USA

## Abstract

Subcapsular liver hematoma (SLH) is a rare complication of HELLP (hemolysis, elevated liver enzymes, low platelets) syndrome. We report a previously healthy 16-year-old female presenting with pre-eclampsia requiring emergent C-section, who developed immediate postoperative bleeding and abdominal distention. Abdominal computed tomography angiography (CTA) revealed a large encapsulated liver hematoma with active extravasation. The patient was successfully treated with a multidisciplinary approach with medical and surgical management.

## 1. Introduction

HELLP syndrome is a complication associated with pre-eclampsia. The overall incidence of HELLP is 0.2–0.6% of all pregnancies and occurs in 4–12% of cases with severe pre-eclampsia [1]. SLH results from spontaneous bleeding between Glisson's capsule and the liver parenchyma and was first reported as a complication of pregnancy in 1844. It was attributed to the patient wrapping a handkerchief around her body to obtain relief from “gastrodynia” leading to a traumatic rupture [2]. SLH has been reported in less than 2% of pregnancies complicated by HELLP [3]. Incidence of SLH with rupture in pregnancies varies from 1/40,000 to 1/250,000 [4] and is associated with an increased maternal mortality rate of 18–86% [5]. Pathogenesis of SLH is not fully understood; however, it is likely related to microangiopathy developed during pregnancy, combined with endothelial dysfunction resulting in an inappropriate activation of the coagulation cascade [6]. Vascular injury can cause hemolysis and blood flow obstruction due to fibrin deposits in the hepatic sinusoids, which increase liver enzymes and aid in formation of SLH [6]. The right hepatic lobe is the most commonly affected lobe and is involved in 75% of cases [7]. SLH complicating HELLP syndrome should be suspected in patients with signs and symptoms, including anorexia, nausea, vomiting, epigastric or right upper quadrant pain, and dyspnea. Rupture should be suspected in the setting of hemodynamic instability. This case report highlights a patient with Grade IV SLH that was managed in an intensive care unit (ICU) with radiological and surgical interventions.

## 2. Case Report

**Figure 1:**
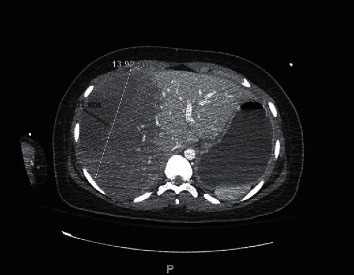
CTA abdomen on admission showing a large right hepatic subcapsular hematoma measuring 14 cm × 3.6 cm in size with active arterial extravasation.

**Figure 2:**
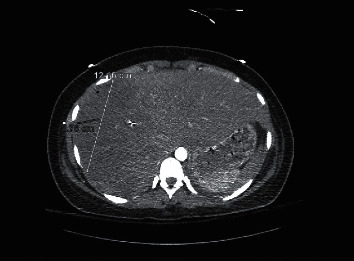
CTA abdomen on postoperative day 4 showing improvement in size and no active arterial extravasation.

A 16-year-old female, gravida 1, para 0, at 36-week gestation presented to the hospital with pre-eclampsia requiring emergent C-section with delivery of twins. Within hours of delivery, the patient was noted to be hypotensive with systolic blood pressure in 70's with slow vaginal bleeding and progressive abdominal distension. One hour after c-section, hemoglobin was 4.5 gm/dL and platelets 58,000/mm^3^. Because of severe anemia and evidence of hypovolemic shock, she was taken to the operating room for an exploratory laparotomy, which revealed hemoperitoneum and a large encapsulated liver hematoma. No other evidence of bleeding elsewhere was noted. She was transfused 4 units (U) of packed red blood cells (pRBC), 2 U fresh frozen plasma (FFP), and 1 U platelets (plt) in the perioperative period. After initial stabilization, she was transferred to our level I trauma center for interventional radiology and further surgical evaluation for possible arterial embolization and hepatic resection if indicated. On arrival, vital signs were remarkable for a heart rate of 104 beats per minute and blood pressure of 156/88 mmHg. The patient was mechanically ventilated and sedated from her initial laparotomy prior to transfer. The lungs were clear to auscultation bilaterally. Her abdomen was open with a wound vacuum in place accompanied by bloody drainage. Laboratory findings revealed aspartate aminotransaminase (AST), 7429 U/L; serum alanine aminotransaminase (ALT), 3345 U/L; serum lactate dehydrogenase (LDH), >12,000 U/L; creatinine, 1.8 mg/dl; white blood cell, 18,700/mm^3^; hemoglobin (Hb), 10.6 mg/dL; platelet count, 79,000/mm^3^; fibrinogen, 173 mg/dL; PT, 16.6 seconds; D-dimer, >3000; and fibrin split product of 10. CTA of the abdomen and pelvis showed a large right hepatic subcapsular hematoma measuring 14 cm × 3.6 cm with active arterial extravasation (Figure 1). Mass effect from the right-sided subcapsular hematoma caused compression of the right hepatic lobe, with associated decreased perfusion. Emergent Gelfoam and coil embolization of the right hepatic artery was performed by interventional radiology. This was subsequently followed by exploratory laparotomy with unroofing of the hepatic capsular hematoma. The patient received another 5 U pRBCs, 2 U FFP, 1 U plt, and prothrombin complex concentrate intraoperatively and was transferred to the intensive care unit (ICU) in critical condition. Given the derangements of liver function, accompanied by thrombocytopenia and an elevated LDH, she was ultimately diagnosed with HELLP syndrome and DIC, complicated by a Grade IV SLH. The patient was extubated on postoperative day 1, and hemoglobin normalized and LFTs started to down trend on day 2. CTA abdomen and pelvis repeated on day 4 showed improvement, and no active extravasation was noted (Figure 2). She was discharged to home on hospital day 7 with long-term follow-up with gastroenterology to monitor liver injury and for repeat imaging of the liver to monitor for resolution.

## 3. Discussion

SLH is a rare and devastating complication of HELLP in pregnancy that requires prompt recognition and a multidisciplinary approach to ensure optimal outcomes. Some risk factors for HELLP include chronic hypertension, multiparity, advanced maternal age, and a previous history of pre-eclampsia or HELLP [8]. In addition, risk factors for pre-eclampsia include a family history, prior history of pre-eclampsia, multiple gestations, and first pregnancy [9]. Symptoms associated with SLH are generally nonspecific and include anorexia, nausea, vomiting, epigastric or right upper quadrant pain, and dyspnea [7]. Rupture may result from trauma precipitated by vomiting, abdominal palpation, transport of the patient, uterine contractions, and manual removal of the placenta [10]. It manifests as hemodynamic compromise and hypovolemic shock. The presence of the abovementioned conditions in a patient with HELLP should raise suspicion for SLH and prompt immediate imaging. Ultrasound is often the first-line modality due to access and availability; however, CT and MRI are both highly sensitive and may facilitate diagnosis as well. Imaging evidence of SLH warrants rapid transfer to a tertiary care center. Medical management is first line for hemodynamically stable patients and encompasses close monitoring of hemodynamic status and coagulation parameters, serial imaging, avoidance of liver manipulation, and replacement of blood products [11]. Alternatively, surgical management including ligation of the portal vein or hepatic artery, partial liver resection, perihepatic packing, and drainage of surgical site [7, 12, 13] should be pursued if rupture occurs and the patient is hemodynamically unstable with signs and symptoms of shock. This in conjunction with radiologic intervention including embolization, and critical care support may also be required in the event of hemodynamic compromise.

## 4. Conclusion

SLH with HELLP syndrome is a rare complication of pre-eclampsia and should be suspected in patients with signs and symptoms including right upper quadrant pain, nausea, vomiting, and anorexia. Rupture should be suspected in the setting of hemodynamic instability. Providers should be aware of the risk factors, signs and symptoms, diagnosis, and treatment options for subcapsular liver hematoma as this condition carries a high mortality if not recognized early and treated appropriately with combined medical, obstetrical, radiology, and surgical team.
